# Milk consumption and behavior of calves in automated calf feeders as early indicators of weaning liveweight

**DOI:** 10.3168/jdsc.2023-0488

**Published:** 2024-03-02

**Authors:** S.W.J. Legge, P.C. Thomson, C.E.F. Clark, S.C. García

**Affiliations:** 1 School of Life and Environmental Sciences, Faculty of Science, The University of Sydney, Camden, NSW 2570, Australia; 2 Sydney Institute of Agriculture, The University of Sydney, Camperdown, NSW 2006, Australia; 3 Sydney School of Veterinary Science, Faculty of Science, The University of Sydney, Camden, NSW 2570, Australia

## Abstract

Modern intensive dairy farming relies on data to aid and prioritize management decisions made on farm. Decisions made early in an animal's life can have lasting effects on welfare, productivity, longevity, and profitability. Precision technology such as automated calf feeders (ACF) allow the customization of feeding programs, but despite this, weaning weights (WWT) vary substantially between calves. This observational study used a 3-yr dataset comprising 1,440 female Holstein Friesian calves at a single intensive commercial dairy farm (Dairy Australia feeding system 5; indoor, total mixed ration) using ACF to (1) determine the variability in WWT (as a proxy of animal performance) of calves within this system; (2) identify the contributing factors responsible for the variation in WWT; and (3) identify potential early management intervention points that could be indicative of the performance of calves at weaning within the system. Calves entered the ACF at 10 d of age with 12 calves per ACF; calves were weighed at birth and weaning using weigh scales. We discovered a large range of calf WWT (41–118 kg/head) at ~60 d of age despite the application of strict uniform management protocols. Our results from modeling showed that WWT was significantly and positively associated with birth weight (BWT), with low BWT calves (<36 kg) achieving an average of 70 kg weight at weaning. In contrast, heavier BWT (>36 kg) calves achieved an average of 82 kg at weaning. Based on calf feeder data, cumulative milk consumption and cumulative unrewarded visits to the feeder, as well as BWT, were identified as indicators of greater WWT as all these were highly significant terms in the model for WWT. Results suggest that quantifying consumption and number of visitations to the ACF at d 5 may allow farmers to identify, with time to intervene, calves underperforming within the feeder or system, therefore increasing their potential for growth. Additionally, this demonstrated that greater milk consumption (>30 kg) and interaction with the feeder up to d 5 in the feeder is more likely to yield a WWT >75 kg, identifying a potential point for management intervention for calves below consumption and interaction thresholds (e.g., by developing alarm systems based on consumption or visitation number).

## Highlights

• Weaning weight among calves showed considerable variability, ranging from 41 to 118 kg.
• Milk consumption and number of visits are strong indicators of performance.
• Intervention points could be effective as early as 5 days.

## Graphical Abstract

**Figure:**
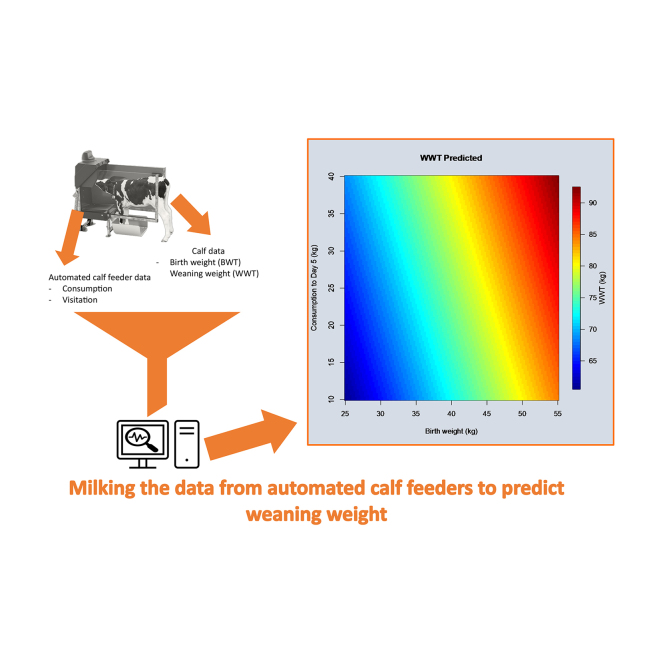
**Summary** This study suggests that by monitoring calves' consumption and visitation frequency using automated calf feeders on day 5, farmers can identify underperforming calves early, allowing for timely intervention to boost their growth potential. Additionally, calves exhibiting higher milk consumption and increased interaction with the feeder during the first 5 days are more likely to achieve a weaning weight exceeding 75 kg. This highlights an opportunity for management intervention in cases where calves fall below these consumption and interaction thresholds.

Automated calf feeders (**ACF**) are an alternative to manual milk feeding. Computer-controlled systems control the amount of milk offered and typically are associated with housing calves in groups while providing individualized feeding of milk. They provide a method to rear calves in groups, especially in intensive systems, by allowing variable amounts of milk to be fed individually several times a day, in some circumstances resulting in an improved rate of weight gain and welfare (Fujiwara et al., 2014) and reducing health-related issues (Jensen, 2003). Automatic calf feeders record the time and number of visits to the feeder, whether the visit was rewarded with milk, the total time spent feeding and total milk consumption for that visit, and the speed of milk consumption in milliliters per minute. However, these data have been predominantly used for the surveillance of calf health (Lowe et al., 2019) rather than to identify factors affecting individual weaning weight (**WWT**) variability, or to estimate future productivity or key points of intervention for calves that are underperforming in terms of daily live weight gain. The performance and growth of calves are known to have an effect on future productivity (Heinrichs and Heinrichs, 2011). Anecdotal evidence suggests that the variability among individual calves (within a group or pen) in their WWT is considerable, although such variability has not been properly quantified through research. Thus, there is a clear gap in knowledge, and therefore an opportunity to explore the potential of the data generated by ACF to quantify individual animal variability, identify factors affecting it, and assess the value of data as a predictive tool of animal performance. The objectives of this cohort observational study were to (1) determine the variability in WWT of animals within this system; (2) identify the contributing factors responsible for the variation in WWT; and (3) identify potential early management intervention points that could be indicative of the performance of calves at weaning within the system.

Ethical approval was not needed as the study was an observational study and only routine animal husbandry procedures were performed. Preweaning calf data from February 2017 through June 2019 were used from an intensive commercial dairy farm milking 1,200 dairy cows in New South Wales, Australia, which operated under strict uniform management to Australian standards. The farm protocol for managing newborn calves was as follows: the calf was removed from the dam as soon as the calf was found, and navels were dipped with 7% iodine. Calves were fed 4 L of colostrum as soon as possible following birth. A second feeding of colostrum of similar quantity and quality was provided approximately 12 h following the first feeding. If a calf did not consume colostrum, an esophageal tube was used. Colostrum quality was assessed using a Brix refractometer. Fresh colostrum was fed if the Brix score was ≥23%; otherwise, frozen colostrum (with a Brix score ≥23%) or colostrum replacer was fed. Blood samples were taken at 4 d of age and serum protein measurements obtained to ensure that the calves had effective immunoglobulin transfer. All calves included within this study were confirmed as having sufficient transfer of passive immunity at levels before inclusion to the study (approximately equivalent to serum total protein IgG levels >10 g/L).

Calves were then placed in individual hutches for the following 10 d and offered 4 L of 26% CP and 21% crude fat milk replacer at 135 g/L (MaxCare Premium, Maxum Foods Pty Ltd.) twice daily through teat buckets with ad libitum water in a bucket and access to oat chaff. At 10 d of age, calves were transferred to the group housing location, which contained Holm and Laue 100 automated calf feeders and maintained the concentration of milk replacer (Holm and Laue, Germany). The group housing area had finely chopped wood chips for bedding, automatic calf brushes (DeLaval, Sweden), overhead fans with misting systems that respond automatically to environmental changes, ad libitum calf starter (CopRice, Australia), and water in troughs accessible through head bales beside the ACF, as well as being in a well-ventilated location with an open-sided shelter design. Stocking density was 12 calves per feeder and a maximum 10-d age difference. If a calf failed to drink within the first 24 h of being introduced to the feeders, staff would intervene and place the calf into the feeder and encourage it to drink. The milk feeders used a teat-based delivery method to offer milk and had backing gates in place to reduce the ability of other calves to displace the calf using the feeder. The allocation of milk was programmed through a main computer in the calf unit and electronic identification ear tags of each calf were used to identify them as they went into the feeder. All calves were offered an allocation of 8 L per day; if this was exhausted calves were still able to enter and initiate non-nutritive suckling (unrewarded visit). Calves were weighed and weaned 56 ± 4 d after entering the ACF pens. Data were collected from 2 independent systems on farm, Dairy Comp 305 (Valley Agricultural Software, Tulare, CA) and the Holm and Laue calf feeder database (Westerrönfeld, Germany). The Dairy Comp system provided information such as birthdate, birth weight (**BWT**), WWT, and WWT date; these were entered into the system as part of commercial practice after weighing calves using a walk-over weigh scale system (Tru-test Datamars, Switzerland) at birth and weaning. The Holm and Laue system included data sorted into 12-h periods within the calf feeders, and each period included the amount of milk consumed (kg), number of visits to the feeder with available ration and drinking (rewarded), visits without drinking (unsuccessful), visits without available ration (unrewarded), half-day milk consumption average (**AHDC**), and average milk consumption speed (mL/min) for that 12-h period. Although our analysis uses the 12-h period or “half-day” data, discussion will refer to whole day values for clarity.

Data were collected from 2,263 female Holstein Friesian calves from the DairyComp software (DairyComp 305, Valley Ag Software, Tulare, CA). Individual calf data from both systems were matched using VLOOKUP in Microsoft Excel (Microsoft Corp.) to establish the total number of complete calf entries. A calf entry was deemed complete if it was successfully tracked from birth to weaning with a complete set of calf feeder data and a clear entry into the group pens with serum IgG levels >10 g/L. A single mismatch at any point in time was considered as a potential risk of inaccurate data and all records for that calf were deleted. After this process, 1,440 calves remained in the dataset for analysis with 45 removed due to IgG levels.

First, to quantify the variability in WWT, all management factors and calf variables (year, season, BWT, WWT, percentage gained, WWT category, and AHDC) and calf feeder variables (drinking speed [measured by a sensor in the ACF], number of unsuccessful visits, rewarded visits, unrewarded visits, and half-day milk consumption) were assessed using descriptive statistics and probability plots to ensure there were no extreme outliers within the data. The descriptive analysis of the data enabled further cleaning of the dataset to remove outliers and determine that splines would be required for modeling. Outliers were classified as an animal that would not be biologically possible, such as a calf with an extreme weight but no consumption data; these were removed at the same time as calves that did not match up in both systems.

To identify factors contributing to the variability in WWT, the following linear model was fitted to the WWT data:

[1] WWT = β_0_ + Year + Season + Year × Season + *s*(BWT) + *s*(AHDC) + ε,

where WWT is the weaning weight of the calf (kg); Year is a 3-level factor for year of birth (2017, 2018, 2019); Season is a 4-level factor for season of calving (spring, summer, autumn, winter); BWT is the calf's birth weight (kg); AHDC is the average half-day consumption of milk (kg) from entry into the ACF to weaning; and ε is the random error. The *s*( ) functions refer to splines of BWT and AHDC, with 4 knots specified at their quintiles, to allow for possible nonlinear associations with WWT. Model fitting was conducted using the lm function, along with the ‘splines' package, in R Statistical Software (v4.3.2; R Core Team, 2023). However, no nonlinearity for BWT was detected, so a linear term was fitted instead (‘*s*(BWT)' replaced with ‘β_1_BWT' in the above model). Following this, the ‘emmeans' package was used to obtain model-based means (‘least squares means') for seasons, years, and specific values of BWT and AHDC. Assumptions of normality and constant variance were assessed through the use of diagnostic residual plots. Statistical significance was declared at α = 0.05.

A further analysis was undertaken to identify potential early indicators of good or poor performance. This analysis assessed the effect of cumulative consumption as well as cumulative number of unrewarded visits up to d 41 within the feeder system on WWT. This was initially visualized using the raw data and the WWT categories to determine if there was potential for practical use on farm. For this, a series of linear regression models was fitted to the WWT data, one for each half day:

[2] WWT = β_0_ + β_1_BWT + β_2_HDC*_h_* + β_3_NUV*_h_* + ε,

where HDC*_h_* is the cumulative consumption of milk up to half-day *h*, and NUV*_h_* is the cumulative number of unrewarded visits up to half-day *h*. To visualize the effects of these 2 variables across half-days, plots of effect size as well as −log_10_ *P*-values versus half-day were produced. Once the most significant cumulative period was determined (*h* = 10, i.e., 5 d in the calf feeder), the fitted model was explored visually in 2 ways. First, the response surface of predicted WWT versus BWT and HDC_10_ (cumulative half-day consumption to d 5 in the feeder) was evaluated over a fine grid using the emmeans and image.plot functions (‘fields' package in R). Finally, the predicted values were used to calculate the probability of a WWT of at least 76 kg (overall median), for a specified BWT and HDC_10_. This was calculated as the upper-tail probability for WWT ≥76, from a normal distribution with mean being the predicted WWT and the standard deviation being the residual SD from the regression model, calculated using the ‘pnorm' function in R.

The range of BWT was 15 to 63 kg with an average of 38.9 kg. The range of WWT varied from 41 to 118 kg (Figure 1) with an average of 76.2 kg demonstrating the large physical size differences between calves at weaning.

**Figure 1:**
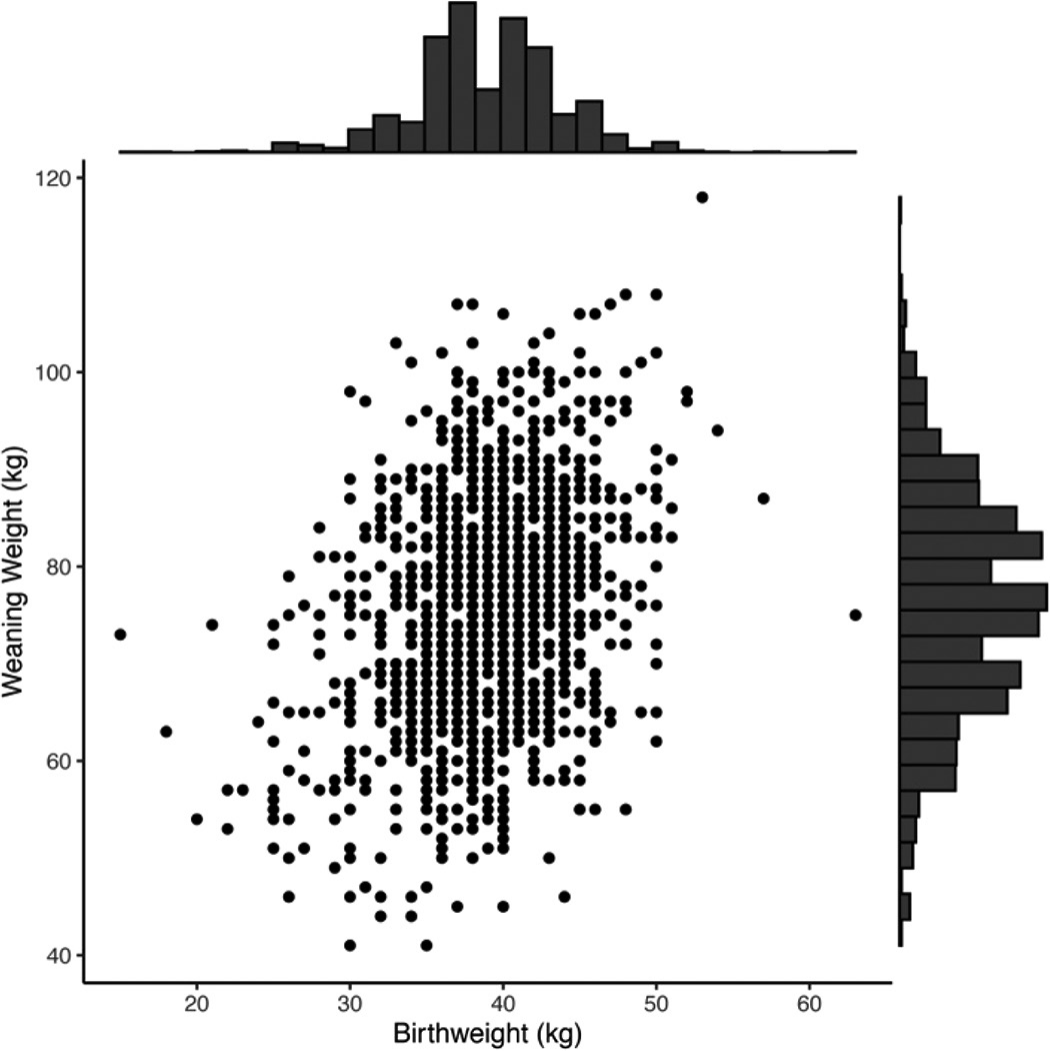
Scatter plot of birthweight (BWT, kg) versus weaning weight (WWT, kg) of 1,440 calves reared within automated calf feeders (ACF) from 2017 to 2019, together with histograms of their individual distributions, shown in the figure margins. Pearson correlation between BWT and WWT is 0.34.

The results of the regression model in Equation 1 indicated that the predictors explained ~29% of the variation in WWT (R^2^ = 0.287, *P* < 0.001). It was found that all terms significantly affected WWT, namely BWT (*P* < 0.001), AHDC (*P* < 0.001), Year (*P* < 0.001), Season (*P* = 0.002), and a significant interaction between Year and Season (*P* < 0.001). There was a strong linear relationship between BWT and WWT (Figure 2). However, this model shows an interesting association between milk consumption (L/half-day per calf) and WWT (Figure 2). The results indicate little or no effect of level of milk consumption on WWT between ~5 and 6 L/d, but WWT increased approximately linearly when calves increased consumption from ~6 to ~8 L/d (~3 to 4 L/half-day). Calves consuming less than 2.25 kg and above 4 kg were very few, which is why the SE fans out at either end of Figure 2.

**Figure 2:**
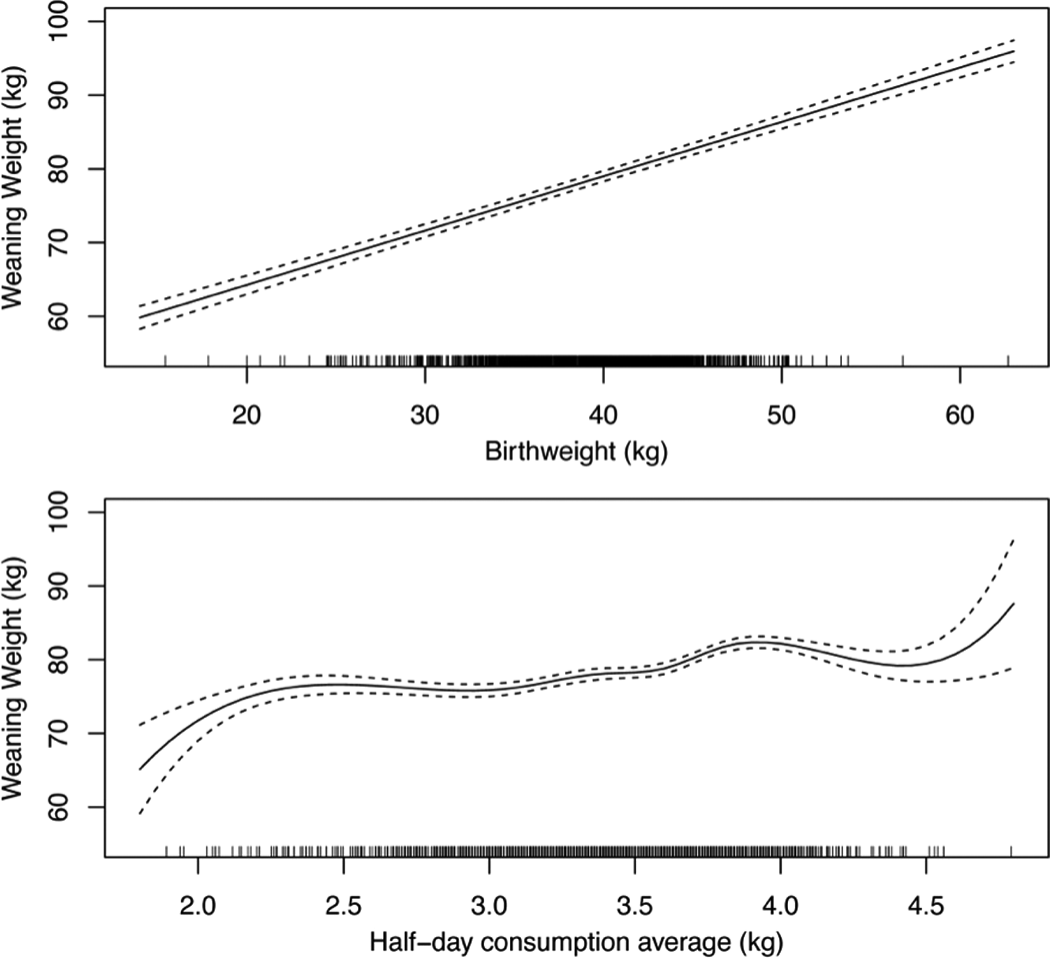
The relationship between weaning weight (WWT, kg) and birth weight (BWT, kg), and average milk consumption (kg), after accounting for the effects of year, season, and either AHDC (for BWT) or BWT (for AHDC), from statistical model [1]. Dotted lines indicate ±SEM.

Cumulative consumption of milk was significant in estimating the WWT performance. While the greatest effect was at d 2, d 5 (i.e., half-day 10) in the feeder was the earliest point where intervention could be made on farm in estimating the potential WWT by consumption of milk (*P* < 0.001), as the reduced SE by this time would result in a more accurate prediction. Cumulative unrewarded visits were also highly significant in predicting WWT (*P* < 0.001) from d 1 to 10 in the feeder. The effect of cumulative unrewarded visits initially peaked after 10 d in the feeder (data not shown). Using the outcome from the model [2], a matrix was created to visually demonstrate the potential WWT for each specified BWT and cumulative consumption of milk up to half-day 10 (HDC_10_) (Figure 3). This shows that, as early as d 5 in the feeder, low BWT calves (BWT 15–36 kg) are likely to only reach ~65 kg/head on average at weaning and demonstrates a trend in which low BWT calves have better outcomes with greater consumption. The graph also shows that heavier BWT calves (BWT >40 kg) will have a better outcome estimated at this point, achieving a mean of no less than ~85 kg/head at weaning.

**Figure 3:**
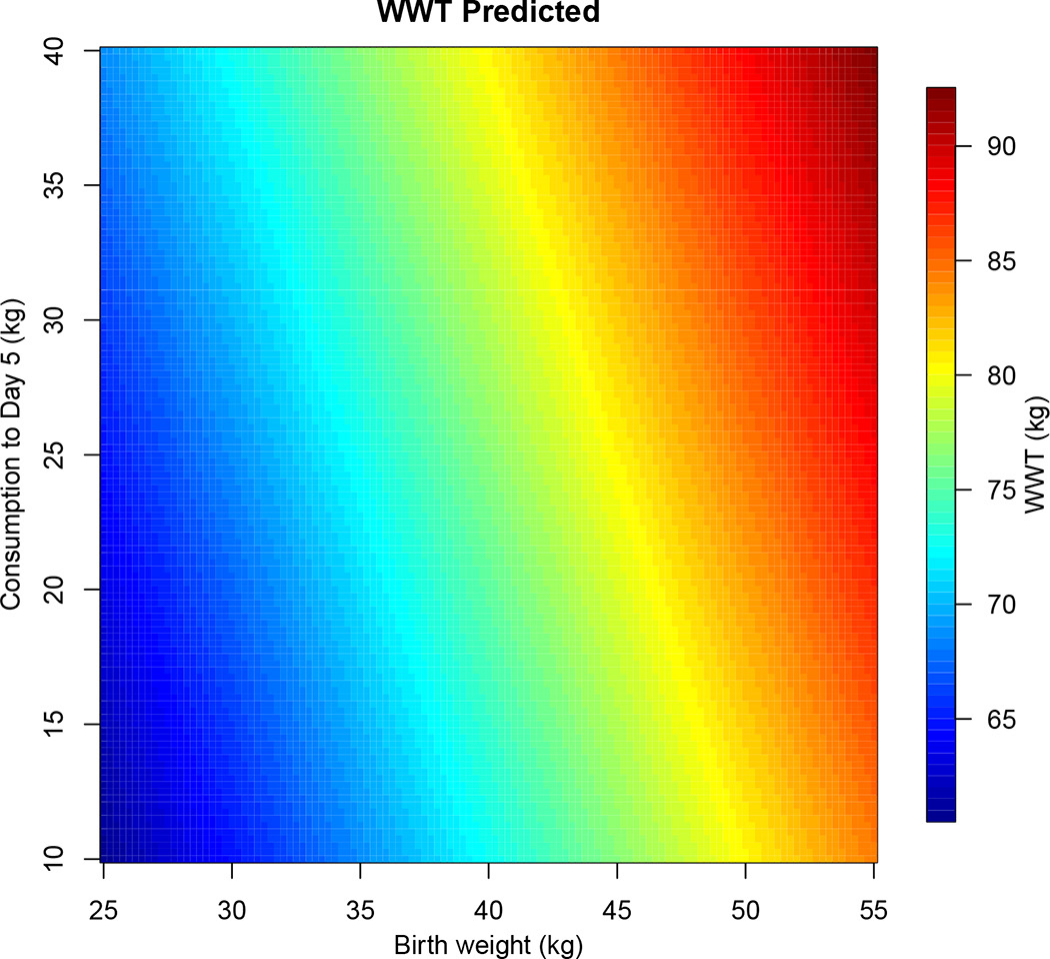
Predicted response surface showing the model-based mean weaning weight (WWT) for varying birth weights and cumulative consumption up to d 5. A scale is indicated on the right side of the plot.

The first and second objectives of this study were to quantify interanimal variability in WWT and identify factors affecting it. Weaning weights ranged from 41 to 118 kg. The factor most associated with WWT was BWT with heavier calves at birth being heavier at weaning. The association between WWT and milk consumption was linear only between the most commonly used commercial range of 6 to 8 kg/d followed by a nonlinear trend of milk consumptions greater than this. Although milk or milk substitute consumption is often identified as the most significant factor for calves in the first 3 wk of life in the literature (Jensen, 2006; de Passillé et al., 2011; Rosenberger et al., 2017), this study demonstrates that milk consumption alone is not responsible for the performance of calves. Davis Rincker et al. (2011) showed calves that consumed greater rations of a milk replacer were heavier and taller at weaning, with these heifers conceiving and calving approximately 15 d earlier than those offered a conventional diet (Davis Rincker et al., 2011). This latter study on milk consumption shows a focus on growth rate through nutrition rather than accounting for the variability stemming from management factors. Within our study, a deeper analysis was needed into the WWT and milk consumption behavior of calves within the dataset to assess if there were any significant differences between the high and low performing animals. The initial analysis (Equation 1) revealed the differences between calves' consumption of milk and BWT and the impact on WWT; the further impact of this on lactation performance is unknown and should be investigated to determine if season of rearing has lasting implications on dairy cattle at this commercial farm.

Another objective of this study was to identify early points in time that could be indicative of future animal performance. Cumulative unrewarded visits showed high levels of differentiation between WWT categories. In agreement with Benetton et al. (2019), self-determined step-down weaning showed that calves with high levels of unrewarded visits were likely to wean at greater weights with the same calves also more likely to consume solid feed more readily (Benetton et al., 2019). As our work did not quantify the consumption of solid feed, future work with ACF should include the behavior toward ACF as well as solid feed to completely understand the performance of calves. Nevertheless, our results imply that application of intervention points could be possible on commercial farms by altering the available milk or milk substitute allocation for a calf that is achieving very high levels of unrewarded visits combined with a low level of unsuccessful visits. A calf that is not meeting these performance indicators could be intervened with to aim to achieve a greater WWT performance. Interventions might be undertaken such as encouraging feeding by farm staff and segregation of calves not meeting their full allocation of milk.

Behavior toward calf feeders has been used to detect illness and disease before clinical symptoms (Sutherland et al., 2018; Lowe et al., 2019) and to predict weaning age (Neave et al., 2019). Other work has shown a negative correlation between latency to drink unassisted from the automated feeder and milk intake in the first 2 wk (Fujiwara et al., 2014) and weight gain in the first month (Medrano-Galarza et al., 2018). Calves that struggle in ACF could be considered slow learners and may be less capable when it comes to maximizing milk intake during the milk feeding period. This could directly affect the transition to solid feed intake in the later stages of weaning, resulting in a lower performance and reduced WWT. Calves may also have difficulty learning from social cues of their conspecifics about their feeding environment, as the development of feeding behaviors can be enhanced through social facilitation and learning from other members of the group (Neave et al., 2019). Our study used milk (substitute) consumption over time to identify the performance of calves, potentially creating an intervention point from these early indicators of WWT performance.

When milk consumption data were combined with BWT data, the WWT was most affected during d 3 to 20 in the calf feeders. This was likely due to the lack of development of the rumen and the reliance on the consumption of milk (Khan et al., 2011). During this period, the number of unrewarded visitations and milk consumption level was highly associated with WWT. Our study suggests that it would be sufficient evidence for the farmer to intervene after calves have spent the first 5 d on the feeder. The implication from these results for on-farm application is that if a calf is struggling to consume (a minimum/given amount of) milk as early as d 5 on the feeder it is likely to underperform (i.e., to achieve a lower WWT). This disadvantage is increased for calves with a lower BWT. Thus, early intervention for calves with relatively lower consumption levels could significantly improve their WWT. This study also addressed the probability of calves reaching the target WWT. This was less likely for lighter BWT calves with low levels of milk consumption and the reverse for heavier BWT calves. A future area worth exploring is the health and incidence of disease for lighter BWT calves because they are identified as a group more likely to require intervention. Literature supports that health and growth are linked and the inclusion of health data in future studies would be beneficial (Heinrichs and Heinrichs, 2011; Medrano-Galarza et al., 2018).

While intervention for performance has not been implemented on farm, intervention for disease has been utilized by using the data from calf feeders and rectal temperature (Sutherland et al., 2018; Lowe et al., 2019). The ability to address calf performance using data from ACF in real time without manual handling presents an opportunity for enhanced calf management as well as improved outcomes on farm. Performance-based intervention such as increased staff interaction and training to the feeder for calves not achieving predetermined thresholds. Further research into intervention periods based on the results of this study would demonstrate the effectiveness of intervention. An additional study into the effect of calf performance on their performance as mature animals would also demonstrate the necessity to harness all available data on farm for improved productivity and profitability.

This study has identified potential intervention points for the improved calf management on farm after 15 d of life (5 d on the ACF) as well as quantifying the variability in WWT. This study also demonstrated that the variability in WWT was associated with both management factors and behavior within the calf feeder, in particular the number of unrewarded visits between d 3 and 10. Also, the cumulative consumption of milk at d 5 in the calf feeder could be used to estimate the WWT of calves when assessed with their BWT. Overall, our study provides the basis for early alerts systems to be developed. Future research incorporating solid feed intake in the analysis of growth alongside the impact of this growth on lifetime performance and health is required.
